# Establishment of Comprehensive Indicators in TCM Pectoral-qi Case Report Based on Experts Diagnosis and Self-test Technology

**DOI:** 10.1097/MD.0000000000009916

**Published:** 2018-02-16

**Authors:** Hao Cheng, Danhui YI, Jiesheng Si, Jingqing Hu, Yan Yang, Jin Peng

**Affiliations:** a National Academy of Innovation Strategy, China Association for Science and Technology; b School of Statistics, Renmin University of China, Beijing, China; c Mailman School of Public Health, Columbia University, New York, NY; d Center for Statistical Consultation; e Center for Applied Statistics, Renmin University of China, Beijing; f College of Economics, Hangzhou Dianzi University, Hangzhou; g Institute of Basic Theory for Chinese Medicine, China Academy of Chinese Medical Sciences, Beijing, China.

**Keywords:** Comprehensive indicator, experts diagnosis, self-test, PLS-SLVM

## Abstract

**Rationale:**

To establish TCM Pectoral-qi comprehensive indicators and highlight the inner structure among different variables in an objective way, the article uses Partial Least Square Second-order Latent Variable Model (PLS-SLVM) and accomplishes 3 different comprehensive indicators based on both experts diagnosis and self-test data. SLVM includes a measurement model that defines the relationship between observed variables and latent variables and a structure model that imputes relationships between latent variables. The article focuses on PLS as the estimation method. Without normal distribution and independence assumptions, PLS uses objective weighting methods based on the data. Bootstrap method (B = 200) is used to calculate the mean value and standard errors of the PLS estimates. The article chooses the percentile interval to obtain the confidence interval of PLS parameters.

**Patient concerns:**

The patients were diagnosed by the means of experts diagnosis and self-test technology. On the one hand, the patients want to know the effect of self-test by wearing a kind of instrument. On the other hand, we want to establish TCM Pectoral-qi comprehensive indicators and highlight the inner structure among different variables in an objective way.

**Diagnoses:**

The group of 59 subjects are the same no matter whether they were diagnosed through TCM Pectoral-qi Assessment Questionnaire of self-test technology.

**Interventions:**

The same group of 59 subjects keep wearing the instrument for hours and get the self-test data consequently.

**Outcomes:**

As one of comprehensive indicator establishing methods, PLS-SLVM highlights the structure state among variables and improves the evaluation efficiency. Furthermore, it provides a new tool and method in TCM diseases prevention and health security.

**Lessons:**

As expected, PLS-SLVM is a useful tool due to its nonassumption of normal distribution and independence with consideration of correlation among different variables. Thus PLS-SLVM can be applied in ordinal data from assessment questionnaire and continuous data about physicochemical indexes for the same group of people. It displays that PLS-SLVM builds a connection between TCM experts diagnosis and the self-testing technology.

## Abstract 2

Supplemental Digital Content is available in the text

The following table is about all the abbreviations of both latent variables and observed ones.

**Figure:**
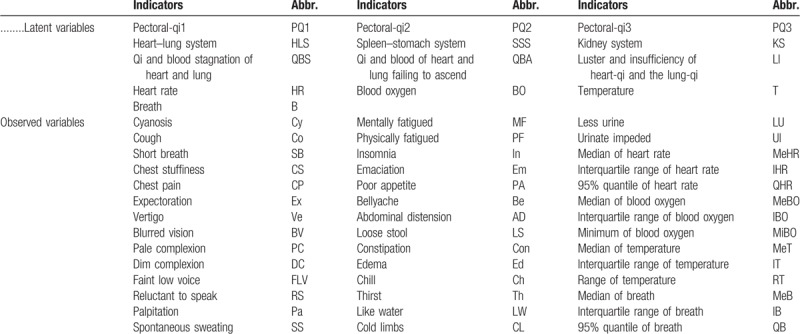


## 1 Introduction

Traditional chinese medicine (TCM) pectoral-qi is transformed from cereal nutrient, accumulates in the chest and works with the breathing air. Language, sound, smell, and breath are all relevant to pectoral-qi. It also maintains circulation of qi and blood supporting heart movement, body temperature, and so on. It is a complex and abstract concept that cannot be measured directly. Thus, it becomes a challenge to establish 1 comprehensive indicator or more to define what is TCM pectoral-qi in an understandable way and how to assess it. Saisana et al^[1]^ definite that a comprehensive indicator is a mathematical combination of single indicators representing different dimensions. Simple linear addition seems an easy way to solve the problem. However, one of its shortages lies in the subjective weighting method that may lead to an unrealistic or idealized structure within the comprehensive indicator. It seems that all the variables have the same contribution in establishing comprehensive indicator. Other subjective weight determination methods are not enough to reflect the objective relationship among variables. Instead, we consider establish a comprehensive indicator in an appropriately statistical way and accomplishing comprehensive evaluation.^[2,3]^

It is not the first attempt to use second-order latent variable model (SLVM) in TCM syndrome evaluation,^[4]^ which is constructed by the measurement model (1) and the structure model (2).^[5,6]^ Here, we denote *x*_*jh*_ as observed variables, *ξ*_*j*_ as first-order latent variables and *η* as a second-order latent variable. *λ*_*jh*_ denotes loading coefficients and *β*_*j*_ denotes path coefficients. Both *ε*_*jh*_ and *δ*_*j*_ are random measurement errors. 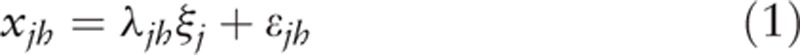 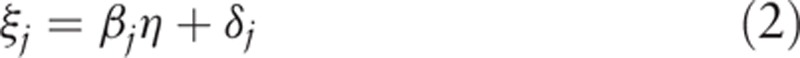


In this article, we also consider Partial Least Square Second-order Latent Variable Model (PLS-SLVM). PLS-SLVM does not need to assume normal distribution and independence among variables. It also calculates objective weight with real data.^[7–10]^ Above all, PLS-SLVM shows obvious advantages compared with other estimating methods especially in dealing with small sample sizes. This is because with small sample sizes, there does not exist identification issues in PLS-SEM (Partial Least Square Structural Equation Model) and PLS-SEM generally achieves high levels of statistical power.^[7]^ Moreover, SLVM is just one kind of structure equation model. Thus, it has the same effect on PLS-SLVM.

Furthermore, we use experts diagnosis results based on both professional assessment questionnaire and physicochemical indexes data supported by self-test technology. About the technology, it is a kind of micro-intelligent health monitoring system, which is worn on the forehead. Infrared and near-infrared sensors are applied to the comprehensive detection of human physiological parameters. Infrared sensor is applied to the measurement of the body temperature. According to the infrared radiation of the target, it was converted into a signal like voltage through a certain transformation to measure the temperature. Heart rate and breathing rate can be obtained after extraction and operation of the corresponding information and data, which generated by the method photo plethysmo graph (PPG). The foundation of the pulse oxygen saturation detection is the Lambert–Beer law and light-scattering theory. Based on the difference of the light absorption coefficient between hemoglobin and deoxygenated hemoglobin with oxygen, pulse oxygen saturation can be get by corresponding transformation and table through the most commonly used clinical method called Reflection Method of PPG.

Based on professional assessment questionnaire data and physicochemical indexes data obtained by self-test technology, we finally establish 3 different comprehensive indicators.

The rest of the article is organized as follows. We describe our methodology in Section 2. In this section, we provide the overall view of our comprehensive indicators based on experts’ diagnosis and self-test technology. We also show the procedures of PLS algorithm, especially how to calculate the scores of latent variables and weight among them. In Section 3, we demonstrate the results in TCM pectoral-qi application. Discussions are included in Section 4.

## 2 Methods

### 2.1 Notation

Data1 is supported by Hu Jingqing Pectoral-qi Research Project from China Academy of Chinese Medical Sciences. TCM Pectoral-qi Assessment Questionnaire consists of 30 items in total, they are cyanosis, cough, short breath, chest stuffiness, chest pain, expectoration, vertigo, blurred vision, pale complexion, dim complexion, faint low voice, reluctant to speak, palpitation, spontaneous sweating, mentally fatigued, physically fatigued, insomnia, emaciation, poor appetite, bellyache, abdominal distension, loose stool, constipation, edema, chill, thirst, like water, cold limbs, less urine, and urinate impeded. Each of the items is given a rating from 1 to 5, with 1 being the no and 5 being serious. For example, cyanosis can be divided into no, mild, moderate, heavy, and serious. According to their physical conditions in recent 4 weeks, all the subjects are diagnosed by TCM experts. Finally, 59 elderly individuals will be studied in our research. Data2 involves 4 physicochemical indexes such as heart rate, blood oxygen, temperature, and breath. All of them can be measured through a kind of wearable instrument on heads. The same group of 59 individuals keep wearing the instrument for hours and get the self-test data consequently.

Each items included in data1 will be used as observed variables directly. For data2, we calculate the statistical characteristics of each variable as observed variables. They are median of heart rate, interquartile range of heart rate, 95% quantile of heart rate, median of blood oxygen, interquartile range of blood oxygen, minimum of blood oxygen, median of temperature, interquartile range of temperature, range of temperature, median of breath, interquartile range of breath, and 95% quantile of breath. Here we list all the observed variables in Table 1.

**Table 1:**
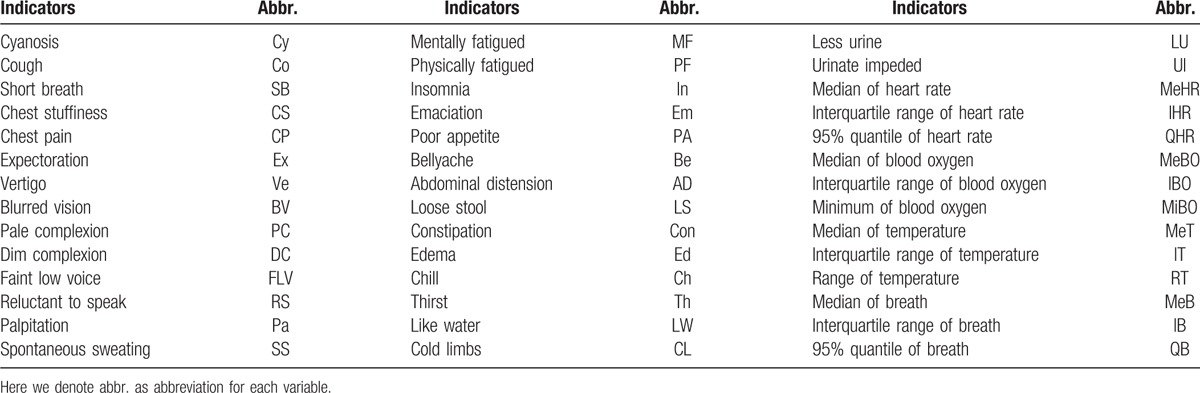
Variables and abbreviations.

### 2.2 Comprehensive indicators design

The common clinical symptoms and signs of the pectoral-qi insufficiency syndrome is the deterioration of the heart–lung function. The cardinal syndromes of common clinical manifestation on pectoral-qi insufficiency syndrome are the weakness of heart–lung function and chaotic circulation of qi and blood, which are in accord with the physiological function of the pectoral-qi insufficiency syndrome known “permeating the heart and vessels to promote circulation of qi and blood.” In addition, spleen–stomach system like poor appetite, loose stool, and abdominal distension alone with kidney system (KS) like edema and inhibited urination can be seen as well. Those are inseparable from the cause of the formation of pectoral-qi insufficiency syndrome. As was described in the book known as *the Integration of Chinese and Western Medicine*, the atmosphere, put the vigor as the basis, the foodstuff essence-qi as nourishment. The formation of pectoral-qi has a tight relation with the vigor and the foodstuff essence-qi. Vigor is generated by kidney and the pectoral-qi is a combination of the foodstuff essence-qi transformed by the spleen–stomach in the thorax and the natural fresh air inhaled by lungs. So it is easier to understand the pectoral-qi insufficiency syndrome commonly has symptoms of dysfunction of the spleen in transportation and impairment of qi transformation due to Yang deficiency. In conclusion, the pectoral-qi insufficiency syndrome is mainly caused by the multiple-organ lesion of the heart, lung in first place and spleen, kidney in second place. Thus, medical researchers and experts demonstrate that all the 30 items of TCM Pectoral-qi Assessment Questionnaire can be divided into 3 dimensions. They are heart–lung system (HLS), spleen–stomach system (SSS), and KS. With all the 3 dimensions, we establish TCM pectoral-qi comprehensive indicator I.

As the monarch of the vigor, the rise and fall of the pectoral-qi has a direct relation with the strength of the heart–lung qi. Deficiency of the heart–lung qi is mainly manifested in pectoral-qi insufficiency. Besides, the syndromes of pectoral-qi insufficiency syndrome are the weakness of heart–lung function and chaotic circulation of qi and blood. Therefore, the cardinal syndromes of common clinical manifestation on pectoral-qi insufficiency syndrome are the failing to circulation qi and blood of heart and lungs, as a consequence, leading to the non-rise of qi and blood of heart and lungs. Therefore, HLS can be divided into 3 more detail dimensions: they are qi and blood stagnation of heart and lung (QBS), qi and blood of heart and lung failing to ascend (QBA) and luster and insufficiency of heart-qi and the lung-qi (LI). Together with SSS and KS, all the 5 dimensions contribute to TCM pectoral-qi comprehensive indicator II.

According to the retrospective study of literature, the pectoral-qi have a certain correlation between the modern medical indexes, for instance, the rhythm of the heart, heart rate, blood pressure, breathing, blood lipid, blood sugar, blood rheology, electrocardiogram, heart function grading, heart function, the brain natriuretic peptide (BNP), the NT-proBNP, plasma angiotensin II, pulmonary function, blood and qi analysis, BODE (body mass index, airflow obstruction, dyspnea, and exercise capacity index) index, and so on. The indicators mentioned above can partly reflect the changes of the pectoral-qi. In the above indicators, the BNP, ultrasound (left ventricular ejection fraction) and electrocardiogram often used to evaluate pulmonary function. Evaluation indicators of pulmonary function mainly include FEV 1% pred, blood and qi analysis (SaO_2_, etc), etc. But indicators, like blood lipid, blood sugar, blood rheology and electrocardiogram, the BNP, ultrasonic, pulmonary function (FEV 1%, BODE index), have disadvantages of insensitive, inspection index with injury and its own factors influence. Heart rate, blood pressure, breathing, blood oxygen saturation, the detection of those 4 indicators related to the pectoral-qi have the advantages of simple operation, noninvasive examination and can be used for remote monitoring in daily life. So these 4 indicators were chosen as the objective indicators in the research of the pectoral-qi. Totally different from the above 2 indicators, TCM pectoral-qi comprehensive indicator III is based on physicochemical indexes data. We establish this indicator from 4 dimensions as follows: heart rate (HR), blood oxygen (BO), temperature (T), and breath (B).^[11,12]^

**Figure 1:**
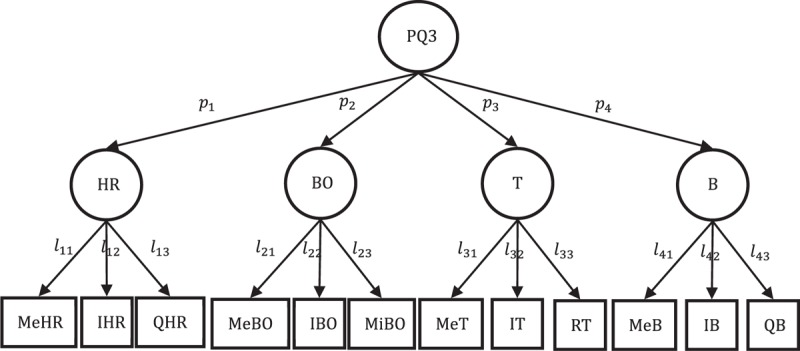
Diagram of comprehensive indicator III. B = breath, BO = blood oxygen, HR = heart rate, IB = Interquartile range of breath, IBO = Interquartile range of blood oxygen, IHR = Interquartile range of heart rate, IT = Interquartile range of temperature, MeB = Median of breath, MeBO = Median of blood oxygen, MeHR = Median of heart rate, MeT = Median of temperature, MiBO = Minimum of blood oxygen, PQ3 = pectoral-qi3, QHR = 95% quantile of heart rate, RT = Range of temperature, T = temperature, QB = 95% quantile of breath. In addition = we denote {*p*_*i*_, *i* =1,2,3,4} as path coefficients and {*l*_*ij*_, *i* = 1,2,3,4, *j* = 1,2,3} as loadings.

We denote PQ1, PQ2, and PQ3 as TCM pectoral-qi comprehensive indicator I, II, and III, respectively. The study has been approved by institutional review boards. They are Institute of Basic Theory for Chinese Medicine and China Academy of Chinese Medical Sciences. All the comprehensive indicators and their corresponding observed variables are showed in Table 2. Taking PQ3 as an example, we show the total structure of a comprehensive indicator in Figure 1.

**Table 2:**
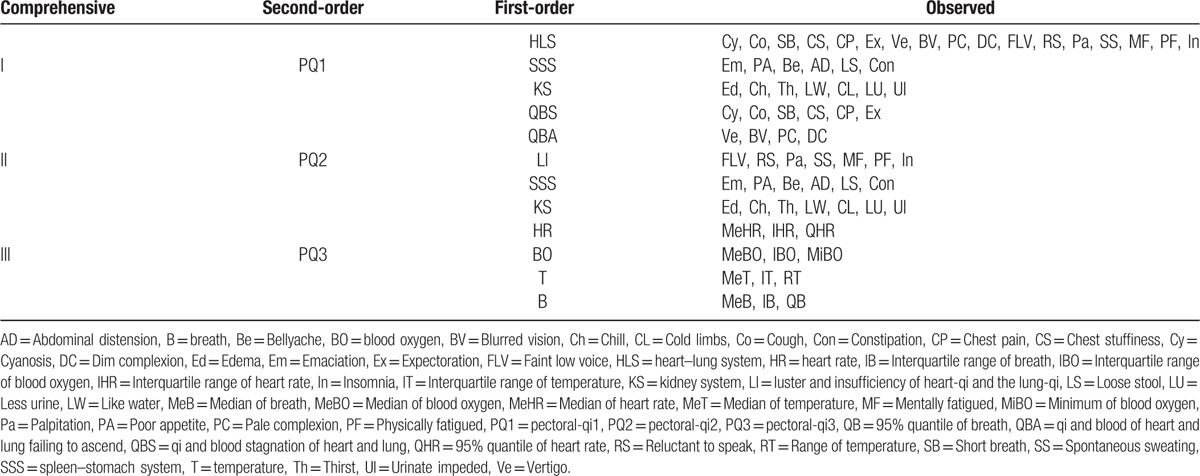
Comprehensive indicators and their corresponding observed variables.

### 2.3 PLS algorithm

To avoid the joint normal distribution and independence assumptions, factor scores and coefficients can be estimated through the PLS method.^[13–15]^ With small sample sizes, PLS-SEM does not have identification issues and generally achieves high levels of statistical power.^[7]^ Thus, PLS is a valuable tool if we only have small amount of sample for SEM or SLVM. The PLS algorithm is as following.

1) First-order latent variables’ outer estimation *Y*_*j*_. The linear combination of centralized observed variable is used to approximate the first-order factor scores. Standardized first-order latent variable with mean 0 and variance 1 can be estimated by Eq. (3). 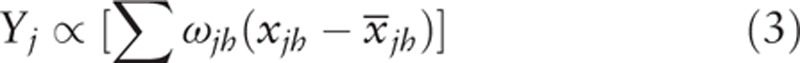  *ω*_*jh*_ is called outer weights with any initial value and generally set to 1.
2) Second-order latent variable's inner estimation *Z*. According to the correlation coefficients between factors, first-order latent variables’ outer estimation can be adjusted constantly. And, the inner estimation can be computed by Eq. (4). 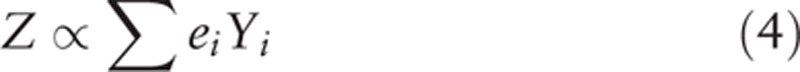  *e*_*i*_ is called inner weights by centroid method.
3) Weights update. Outer and inner weights are updated by Eqs. (5) and (6), respectively. 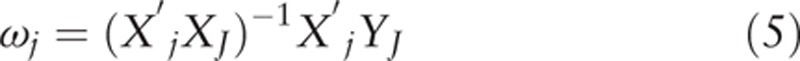 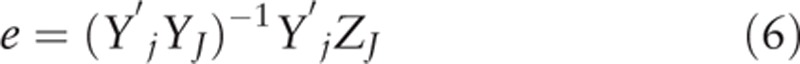

Repeat the above steps until convergence, where the common convergence criteria are that the difference between 2 adjacent iterations is less than 10^−5^.

## 3 Results

**Figure 2:**
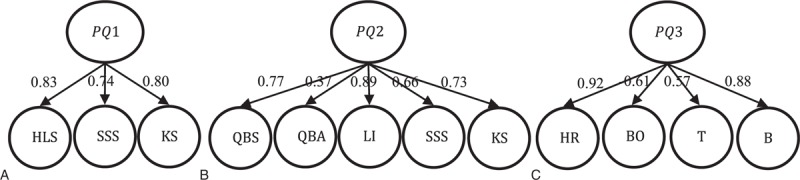
Diagram of TCM pectoral-qi index model with estimated path coefficients. B = breath, BO = blood oxygen, HLS = heart–lung system, HR = heart rate, KS = kidney system, LI = luster and insufficiency of heart-qi and the lung-qi, PQ1 = pectoral-qi1, PQ2 = pectoral-qi2, PQ3 = pectoral-qi3, QBA = qi and blood of heart and lung failing to ascend, QBS = qi and blood stagnation of heart and lung, SSS = spleen–stomach system, T = temperature.

Without consideration of independence assumption, PLS-SLVM illustrates the objective relationship among variables. In the outer estimation procedure of PLS algorithm, the correlation coefficients between observed variables and first-order latent variables and latent variables themselves will be calculated. The magnitude and sign (positive and negative) of the correlation coefficients work on each iteration of outer estimation. Constant inner and outer adjustments lead to repeated updates of inner weights and outer weights until convergence. As one of the important estimation results, path coefficients quantify the relationship between second- and first-order constructs, highlighting the structure state of the comprehensive indicator. With layout of different latent variables and unidirection association, Figure 2 illustrates 3 TCM pectoral-qi comprehensive indicators with estimated path coefficients. Loadings and their bootstrap estimates can be seen in Appendix.

The magnitude and sign (positive and negative) of path coefficients suggest the relationship direction and levels between second-order latent variables and first-order latent variables in structure model. The sign of path coefficients shows whether the relationship between second-order latent variable pectoral-qi and first-order latent variables in different models is positive or negative. In TCM pectoral-qi comprehensive indicators I and II, there exists positive relationship between second-order latent variable pectoral-qi and first-order latent variables (Figure 2A and B), which demonstrates first-order latent variables contributes on pectoral-qi evaluation positively. In TCM pectoral-qi comprehensive indicator III, second-order latent variable pectoral-qi is positively related with heart rate (HR), temperature (T), and breath (B), and negatively related with blood oxygen (BO) (Figure 2C). It concludes that except for blood oxygen (BO), all the other 3 first-order latent variables have a positive effect on the evaluation of pectoral-qi. The magnitude of path coefficients quantifies different association levels between second-order latent variable pectoral-qi and first-order latent variables. In TCM pectoral-qi comprehensive indicator I, the path coefficient of HLS is the most significant (0.83), followed by KS (0.80) and SSS is the least significant (0.74). It shows that HLS plays the most important role in pectoral-qi evaluation, that is, it contributes most in determining whether the pectoral-qi is sufficient or not. In TCM pectoral-qi comprehensive indicator II, the path coefficient of luster and insufficiency of heart-qi and the lung-qi (LI) ranks the first (0.89), QBS ranks the second (0.77), KS ranks the third (0.73). It shows luster and insufficiency of heart-qi and the lung-qi (LI) affects pectoral-qi most, 2 of the 3 dimensions about heart and lung make a relatively higher contribution on pectoral-qi state assessment. In TCM pectoral-qi comprehensive indicator III, the path coefficient of heart rate (HR) is the largest (0.92), breath (B) is less (0.88), and temperature (T) is the least (0.57), the path coefficients of blood oxygen (BO) below zero (−0.61). The tiny difference between heart rate (HR) and breath (B) in path coefficients (0.04) demonstrates their similar performance on pectoral-qi evaluation. Compared with temperature (T) and blood oxygen (BO), heart rate (HR), and breath (B) contributes more on pectoral-qi state assessment.

Table 3 shows the raw estimations, mean biases, standard errors, and confidence intervals of the estimated path coefficients based on TCM pectoral-qi comprehensive indicators from 200 bootstrap method. Raw estimations are the point estimates based on real data before bootstrap. Mean biases mean the difference between mean value of the estimated path coefficients from 200 bootstrap and raw estimations. Standard errors are also from 200 bootstrap. Here confidence intervals are percentile intervals (*α* = 0.1), which is a kind of bootstrap confidence interval. *α* = 0.1 means the 10% percentile of 200 bootstrap replications, which is the lower limit of the confidence interval. 1–*α* = 0.9 means the 90% percentile of 200 bootstrap replications, which is the upper limit of the confidence interval. It indicates that the mean biases and standard errors of the estimated path coefficients are relatively small. Table 3 shows that PLS algorithm used in SLVM helps to get stable and reliable estimations.

**Table 3:**
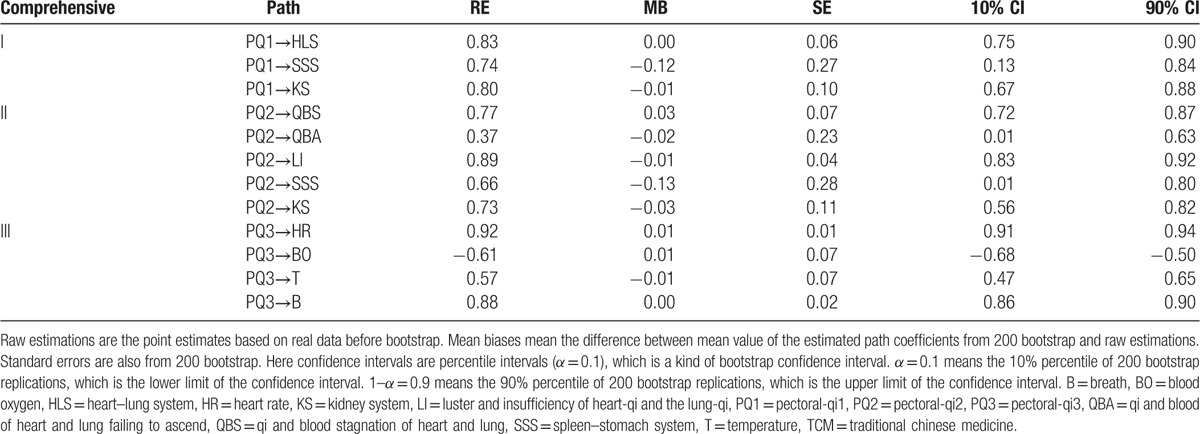
Raw estimations (RE), mean biases (MB), standard errors (SE), and confidence intervals (CI) of the estimated path coefficients based on TCM pectoral-qi comprehensive indicators from 200 bootstrap method.

All the research above demonstrates that heart and lung related dimensions have more effect on pectoral-qi evaluation no matter which TCM pectoral-qi comprehensive indicator we choose. In TCM pectoral-qi comprehensive indicator III, we have HLS. In TCM pectoral-qi comprehensive indicator II, we have QBS, QBA, and luster and insufficiency of heart-qi and the lung-qi (LI). In TCM pectoral-qi comprehensive indicator III, we have heart rate (HR) and breath (B). The commonness in heart and lung aspects in comprehensive indicator design builds a foundation for further pectoral-qi assessment investigation.

Different from TCM pectoral-qi comprehensive indicators I and II, III does not need TCM experts diagnosis through a professional questionnaire. Instead it bases on one kind of wearable instrument to test the subjects’ physicochemical indexes on their own. By comparison, the first 2 indicators fully depend on the TCM experts, together with high time cost and complex operations. Despite that model II has less items than model I for first-order latent variables averagely, the total number of items have not been reduced yet. That is, there still exist 30 items to be scored for each subject. If the subjects choose self-test technology and evaluate pectoral-qi themselves, limited medical resources will never be a question and the evaluation efficiency of comprehensive indicator can be raised at the same time.

In conclusion, PLS-SLVM not only highlights the structure of comprehensive indicators with consideration of the correlation between different variables, but also builds a relationship between different TCM pectoral-qi evaluation ways. As a new tool in data collection, wearable instrument and self-test technology is more convenient and efficient in TCM pectoral-qi evaluation and related research.

## 4 Discussion

As a comprehensive indicator establishing method, PLS-SLVM breaks through the limitations of normal distribution assumption, independence assumption, and subjective weight determination.^[16]^ Based on the real data, PLS-SLVM takes the objective relationship among variables into consideration, illustrates the structure state of variables through path coefficients and interprets the contents of the comprehensive indicator completely. As a new analysis tool, the PLS-SLVM comprehensive indicator can be used to research for TCM syndrome evaluation. In other words, on the base of finding the similarities in both TCM experts diagnosis and self-test in physicochemical indexes, PLS-SLVM builds a relationship on both sides and evaluates TCM syndrome through self-test by a kind of wearable instrument.

In the article, we apply 3 existed different indicators to evaluate TCM pectoral-qi. Each indicator presents one kind of setting that PLS-SLVM can be used. From TCM pectoral-qi comprehensive indicator I to III, we define them as setting 1, 2 and 3. Setting 1 is when observed variables are ordinal and there exists relatively big difference among the number of observed variables under different dimensions. For example, there are eighteen observed variables under HLS, while there are only 6 or 7 observed variables under spleen–stomach system (SSS) and KS. Setting 2 is when observed variables are ordinal and the number of observed variables under different dimensions are more balanced. Setting 3 is when observed variables are continuous. It demonstrates that PLS-SLVM can handle all these settings and helps TCM syndrome investigation. Therefore, PLS-SLVM will play a role in health management, diseases prevention and TCM treatment.

However, PLS-SLVM cannot be used to solve all comprehensive indicator establishing problems. Only when highly association among first-order latent variables really exists, first-order latent variables focus on one topic at the same level and second-order latent variable can be used to reflect and explain the common factor can the researchers take SLVM into consideration.^[17]^ In certain special cases such as small sample and many parameters to be estimated, PLS-SLVM ensures the model can be identified and simplified. In addition, demographic characteristics need to be considered before establishing a comprehensive indicator. The article takes elderly people as example, confirming the methodology advantages of PLS-SLVM. Whether the method is suitable for other group of people need to be checked for further study.

## Supplementary Material

Supplemental Digital Content
