# Supplementary material for: Establishment of Comprehensive Indicators in TCM Pectoral-qi Case Report Based on Experts Diagnosis and Self-test Technology
Source: Medicine (Baltimore). 2018 Feb 16;97(7):e9916. doi: 10.1097/MD.0000000000009916 (PMC5839871; doi:10.1097/MD.0000000000009916)
Supplement: Supplemental Digital Content [file medi-97-e9916-s001.docx]

## Appendix

Loadings and their bootstrap estimates in TCM pectoral-qi indicator Ⅰ, Ⅱ and Ⅲ

| Ⅰ | | | | | | Ⅱ | | | | | | Ⅲ | | | | | |
| --- | --- | --- | --- | --- | --- | --- | --- | --- | --- | --- | --- | --- | --- | --- | --- | --- | --- |
| Path | R.E. | M.B. | S.E. | 10%C.I. | 90%C.I. | Path | R.E. | M.B. | S.E. | 10%C.I. | 90%C.I. | Path | R.E. | M.B. | S.E. | 10%C.I. | 90%C.I. |
| HLS→Cy | 0.35 | -0.03 | 0.18 | 0.11 | 0.53 | QBS→Cy | 0.36 | 0.00 | 0.21 | 0.07 | 0.60 | HR→MeHR | 0.86 | 0.00 | 0.02 | 0.83 | 0.88 |
| HLS→Co | 0.62 | -0.02 | 0.11 | 0.44 | 0.72 | QBS→Co | 0.76 | -0.02 | 0.08 | 0.64 | 0.84 | HR→IHR | 0.64 | 0.00 | 0.05 | 0.57 | 0.70 |
| HLS→SB | 0.73 | 0.00 | 0.08 | 0.62 | 0.82 | QBS→SB | 0.77 | 0.00 | 0.08 | 0.67 | 0.85 | HR→QHR | 0.97 | 0.00 | 0.01 | 0.96 | 0.97 |
| HLS→CS | 0.79 | -0.01 | 0.06 | 0.71 | 0.84 | QBS→CS | 0.78 | 0.00 | 0.06 | 0.70 | 0.86 | BO→MeBO | 0.84 | 0.00 | 0.03 | 0.81 | 0.87 |
| HLS→CP | 0.51 | -0.01 | 0.14 | 0.31 | 0.70 | QBS→CP | 0.65 | -0.01 | 0.11 | 0.48 | 0.76 | BO→IBO | -0.89 | 0.00 | 0.02 | -0.91 | -0.86 |
| HLS→Ex | 0.32 | -0.04 | 0.17 | 0.04 | 0.49 | QBS→Ex | 0.38 | -0.05 | 0.23 | -0.01 | 0.60 | BO→MiBO | 0.87 | 0.00 | 0.02 | 0.84 | 0.89 |
| HLS→Ve | 0.16 | -0.01 | 0.17 | -0.08 | 0.35 | QBA→Ve | 0.84 | -0.17 | 0.35 | 0.00 | 0.87 | T→MeT | -0.43 | 0.00 | 0.09 | -0.53 | -0.31 |
| HLS→BV | 0.37 | -0.03 | 0.13 | 0.16 | 0.52 | QBA→BV | 0.61 | -0.06 | 0.24 | 0.19 | 0.77 | T→IT | 0.91 | 0.00 | 0.02 | 0.89 | 0.93 |
| HLS→PC | 0.31 | -0.02 | 0.18 | 0.05 | 0.50 | QBA→PC | 0.45 | 0.02 | 0.20 | 0.17 | 0.71 | T→RT | 0.92 | -0.01 | 0.02 | 0.89 | 0.94 |
| HLS→DC | 0.26 | 0.02 | 0.16 | 0.06 | 0.47 | QBA→DC | -0.42 | 0.19 | 0.40 | -0.61 | 0.30 | B→MeB | 0.88 | 0.00 | 0.02 | 0.86 | 0.90 |
| HLS→FLV | 0.63 | -0.01 | 0.12 | 0.47 | 0.76 | LI→FLV | 0.59 | 0.00 | 0.12 | 0.44 | 0.72 | B→IB | 0.78 | -0.01 | 0.04 | 0.71 | 0.82 |
| HLS→RS | 0.63 | -0.01 | 0.12 | 0.47 | 0.75 | LI→RS | 0.68 | 0.00 | 0.10 | 0.55 | 0.80 | B→QB | 0.96 | 0.00 | 0.01 | 0.95 | 0.97 |
| HLS→Pa | 0.50 | 0.00 | 0.14 | 0.31 | 0.67 | LI→Pa | 0.48 | -0.01 | 0.17 | 0.22 | 0.67 | - | - |  |  |  |  |
| HLS→SS | 0.39 | 0.00 | 0.16 | 0.19 | 0.58 | LI→SS | 0.46 | -0.01 | 0.17 | 0.23 | 0.66 | - | - |  |  |  |  |
| HLS→MF | 0.74 | -0.01 | 0.10 | 0.60 | 0.82 | LI→MF | 0.79 | 0.00 | 0.07 | 0.71 | 0.86 | - | - |  |  |  |  |
| HLS→PF | 0.69 | 0.00 | 0.07 | 0.58 | 0.77 | LI→PF | 0.76 | -0.01 | 0.07 | 0.66 | 0.83 | - | - |  |  |  |  |
| HLS→In | 0.23 | -0.01 | 0.16 | 0.00 | 0.43 | LI→In | 0.37 | 0.00 | 0.19 | 0.11 | 0.60 | - | - |  |  |  |  |
| SSS→Em | 0.53 | -0.18 | 0.42 | -0.25 | 0.77 | SSS→Em | 0.53 | -0.20 | 0.45 | -0.48 | 0.78 | - | - |  |  |  |  |
| SSS→PA | 0.73 | -0.19 | 0.40 | 0.00 | 0.86 | SSS→PA | 0.73 | -0.20 | 0.43 | -0.15 | 0.86 | - | - |  |  |  |  |
| SSS→Be | 0.62 | -0.13 | 0.29 | 0.00 | 0.79 | SSS→Be | 0.62 | -0.14 | 0.29 | 0.00 | 0.79 | - | - |  |  |  |  |
| SSS→AD | 0.47 | -0.06 | 0.28 | 0.00 | 0.72 | SSS→AD | 0.47 | -0.05 | 0.25 | 0.00 | 0.70 | - | - |  |  |  |  |
| SSS→LS | -0.18 | 0.10 | 0.31 | -0.47 | 0.34 | SSS→LS | -0.18 | 0.16 | 0.31 | -0.40 | 0.45 | - | - |  |  |  |  |
| SSS→Con | 0.45 | -0.08 | 0.35 | -0.03 | 0.76 | SSS→Con | 0.45 | -0.12 | 0.36 | -0.19 | 0.69 | - | - |  |  |  |  |
| KS→Ed | 0.58 | -0.01 | 0.18 | 0.32 | 0.76 | KS→Ed | 0.58 | -0.02 | 0.18 | 0.30 | 0.76 | - | - |  |  |  |  |
| KS→Ch | 0.59 | -0.02 | 0.15 | 0.36 | 0.73 | KS→Ch | 0.59 | -0.01 | 0.16 | 0.37 | 0.76 | - | - |  |  |  |  |
| KS→Th | 0.46 | -0.04 | 0.25 | 0.04 | 0.72 | KS→Th | 0.46 | -0.02 | 0.24 | 0.10 | 0.72 | - | - |  |  |  |  |
| KS→LW | 0.32 | -0.02 | 0.22 | 0.01 | 0.59 | KS→LW | 0.32 | -0.01 | 0.25 | -0.03 | 0.62 | - | - |  |  |  |  |
| KS→CL | 0.65 | -0.01 | 0.15 | 0.44 | 0.79 | KS→CL | 0.65 | -0.02 | 0.17 | 0.45 | 0.79 | - | - |  |  |  |  |
| KS→LU | 0.51 | -0.02 | 0.22 | 0.20 | 0.71 | KS→LU | 0.51 | -0.04 | 0.21 | 0.20 | 0.71 | - | - |  |  |  |  |
| KS→UI | 0.64 | -0.02 | 0.14 | 0.46 | 0.76 | KS→UI | 0.64 | -0.02 | 0.16 | 0.45 | 0.78 | - | - |  |  |  |  |

Raw estimations are the point estimates based on real data before bootstrap. Mean biases mean the difference between mean value of the estimated path coefficients from 200 bootstrap and raw estimations. Standard errors are also from 200 bootstrap. Here confidence intervals are percentile intervals (
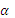

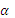
=0.1), which is a kind of bootstrap confidence interval.
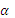

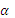
=0.1 means the 10% percentile of 200 bootstrap replications, which is the lower limit of the confidence interval.
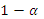

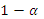
=0.9 means the 90% percentile of 200 bootstrap replications, which is the upper limit of the confidence interval. HLS, heart-lung system; SSS, spleen-stomach system; KS, kidney system; QBS, qi and blood stagnation of heart and lung; QBA, qi and blood of heart and lung failing to ascend; LI, luster and insufficiency of heart-qi and the lung-qi; HR, heart rate; BO, blood oxygen; T, temperature; B, breath; Cy, Cyanosis; Co, Cough; SB, Short breath; CS, Chest stuffiness; CP, Chest pain; Ex, Expectoration; Ve, Vertigo; BV, Blurred vision; PC, Pale complexion; DC, Dim complexion; FLV, Faint low voice; RS, Reluctant to speak; Pa, Palpitation; SS, Spontaneous sweating; MF, Mentally fatigued; PF, Physically fatigued; In, Insomnia; Em, Emaciation; PA, Poor appetite; Be, Bellyache; AD, Abdominal distension; LS, Loose stool; Con, Constipation; Ed, Edema; Ch, Chill; Th, Thirst; LW, Like water; CL, Cold limbs; LU, Less urine; UI, Urinate impeded; MeHR, Median of heart rate; IHR, Interquartile range of heart rate; QHR, 95% quantile of heart rate; MeBO, Median of blood oxygen; IBO, Interquartile range of blood oxygen; MiBO, Minimum of blood oxygen; MeT, Median of temperature; IT, Interquartile range of temperature; RT, Range of temperature; MeB, Median of breath; IB, Interquartile range of breath; QB, 95% quantile of breath.
